# Evaluating atezolizumab in patients with urinary tract squamous cell carcinoma (AURORA): study protocol for a single arm, open-label, multicentre, phase II clinical trial

**DOI:** 10.1186/s12885-023-11397-x

**Published:** 2023-09-19

**Authors:** Simon Crabb, Robin Wickens, Sarah Jane-Bibby, Denise Dunkley, Megan Lawrence, Allen Knight, Robert Jones, Alison Birtle, Robert Huddart, Mark Linch, Jonathan Martin, Adam Coleman, Konstantinos Boukas, Hannah Markham, Gareth Griffiths

**Affiliations:** 1 Southampton Clinical Trials Unit, University of Southampton, Southampton, UK; 2 University Hospital Southampton NHS Foundation Trust, Southampton, UK; 3 Action Bladder Cancer UK (Registered Charity No: 1164374), Tetbury, UK; 4 School of Cancer Sciences, University of Glasgow, Glasgow, UK; 5 Lancashire Teaching Hospitals NHS Foundation Trust, Preston, UK; 6 The Royal Marsden NHS Foundation Trust, London, UK; 7 University College London Hospital, London, UK; 8 Research Department of Primary Care and Population Health, University College London, London, UK; 9 Experimental Cancer Medicine Centre (ECMC), University of Southampton, Southampton, UK; 10 Wessex Investigational Sciences Hub Laboratory (WISH Lab), University of Southampton, Southampton, UK

**Keywords:** Urinary tract squamous cell carcinoma, Atezolizumab, Immunotherapy, Translational research, PD-L1 expression, Phase II

## Abstract

**Background:**

Bladder and urinary tract cancers account for approximately 21,000 new diagnoses and 5,000 deaths annually in the UK. Approximately 90% are transitional cell carcinomas where advanced disease is treated with platinum based chemotherapy and PD-1/PD-L1 directed immunotherapy. Urinary tract squamous cell carcinoma (UTSCC) accounts for about 5% of urinary tract cancers overall making this a rare disease. We have yet to establish definitive systemic treatment options for advanced UTSCC.

Preliminary translational data, from UTSCC patient tumour samples, indicate high PD-L1 expression and tumour infiltrating lymphocytes in a proportion of cases. Both of these features are associated with differential gene expression consistent with a tumour/immune microenvironment predicted to be susceptible to immune checkpoint directed immunotherapy which we will evaluate in the AURORA trial.

**Methods:**

AURORA is a single arm, open-label, multicentre,UK phase II clinical trial. 33 patients will be recruited from UK secondary care sites. Patients with UTSCC, suitable for treatment with palliative intent, will receive atezolizumab PD-L1 directed immunotherapy (IV infusion, 1680 mg, every 28 days) for one year if tolerated. Response assessment, by cross sectional imaging will occur every 12 weeks. AURORA uses a Simon’s 2-stage optimal design with best overall objective response rate (ORR, by RECIST v1.1) at a minimum of 12 weeks from commencing treatment as the primary endpoint. Secondary endpoints will include overall survival, progression-free survival, duration of response, magnitude of response using waterfall plots of target lesion measurements, quality of life using the EORTC QLQ-C30 tool, safety and tolerability (CTCAE v5) and evaluation of potential biomarkers of treatment response including PD-L1 expression. Archival tumour samples and blood samples will be collected for translational analyses.

**Discussion:**

If this trial shows atezolizumab to be safe and effective it may lead to a future late phase randomised controlled trial in UTSCC. Ultimately, we hope to provide a new option for treatment for such patients.

**Trial registrations:**

EudraCT Number: 2021-001995-32 (issued 8^th^ September 2021); ISRCTN83474167 (registered 11 May 2022); NCT05038657 (issued 9th September 2021).

## Background

Urinary tract cancers account for approximately 21,000 new diagnoses and 5,000 deaths annually in the UK [1]. Approximately 90% are composed of transitional cell carcinoma (TCC) histology and are treated with platinum based chemotherapy and PD-1/PD-L1 directed immunotherapy. The approximate incidence of non-TCC urinary tract cancers in the UK is 1.7/100,000/year, and urinary tract cancers may also present with mixed histological components. Urinary tract squamous cell carcinoma (UTSCC) is the most common of the rare urinary tract cancer histologies, comprising between 2.1–6.7% of urinary tract cancers overall. Patients with UTSCC have more advanced disease stage at diagnosis, on average, compared to TCC, with approximately 70% having muscle invasive disease. If confined to the bladder, 5-year disease-free survival following radical cystectomy is 43–57% [2]. UTSCC appears to carry a worse prognosis overall, and on a stage by stage basis compared to TCC [3]. In part, this may reflect more limited, and substantially less researched, treatment options.

Radical cystectomy is the mainstay of treatment for UTSCC, where the disease remains localized [4, 5]. We have yet to establish a definitive systemic treatment approach for UTSCC and it is conventionally considered to respond poorly to chemotherapy. These patients are commonly disadvantaged as a result of explicit exclusion from urinary tract cancer clinical trials. The sole example of a dedicated, prospective, UTSCC clinical trial, to our knowledge, was a single arm phase II clinical trial conducted in the 1990s (BA08) of cisplatin, methotrexate and vinblastine chemotherapy (CMV) for advanced UTSCC. In 38 patients, CMV induced a 9 week objective response rate of 39% with a median overall survival of 7.8 months [6].

Data support the use of PD-1/PD-L1 directed immunotherapy for selected patients with TCC in the first line, maintenance and second line palliative settings [7–12]. Supported by phase III second line and single arm phase II first line data, atezolizumab, avelumab (anti-PD-L1), pembrolizumab and nivolumab (anti-PD-1), are approved for use in Europe in patients with advanced TCC [8–12].

Some immunotherapy trials have accepted UTSCC patients within a wider cohort. The SAUL single arm phase IIIb safety study assessed the PD-L1 targeted agent atezolizumab in 1004 urinary tract cancer patients in the second line, post chemotherapy, setting, but did not provide data relating specifically to outcomes within the UTSCC subtypes that were included [13]. The phase II PURE-01 trial evaluated pembrolizumab, a PD-1 inhibitor immunotherapy, as a neoadjuvant treatment for T2-4a N0 M0 bladder cancer, in 114 patients [14]. Seven of these patients had UTSCC, among whom six had down staging to pT < 1, with one patient achieving a complete pathological response, consistent with a hypothesis predicting clinical efficacy for PD-1/PD-L1 directed immunotherapy in UTSCC.

AURORA is the first dedicated clinical trial of immunotherapy for advanced UTSCC and only the second ever undertaken for systemic therapy. AURORA is part of the International Rare Cancers Initiative (IRCI) Rare Genito-Urinary (GU) Working Group portfolio, and if shown to be a promising treatment in the UK setting the next step will be to develop a randomised international trial to recruit among international partners.

## Methods/Design

The AURORA trial is a phase II trial of atezolizumab immunotherapy, in immunotherapy naive patients with UTSCC.

### Objectives 

The primary objective is to determine the clinical activity of atezolizumab in patients with incurable, histologically confirmed, immunotherapy naive UTSCC.

Secondary objectives are to determine safety and tolerability, overall survival (OS), progression-free survival (PFS), duration of objective response and impact on quality of life of patients treated with atezolizumab. Secondary objectives also include the determination of changes in tumour burden of individual patients and the impact of PD-L1 expression status on clinical response.

The translational research objectives for the study include investigating PD-L1 expression in tumour and immune cell infiltrate, tumour infiltrating lymphocytes percentage, gene expression analysis, intra-tumoral T-Cell receptor repertoire, morphological evaluation of tumour infiltrating immune cells and their spatial distribution, tumour mutational burden, circulating immune cell profiles, and peripheral T-Cell receptor clonality.

### Study design

**Fig. 1:**
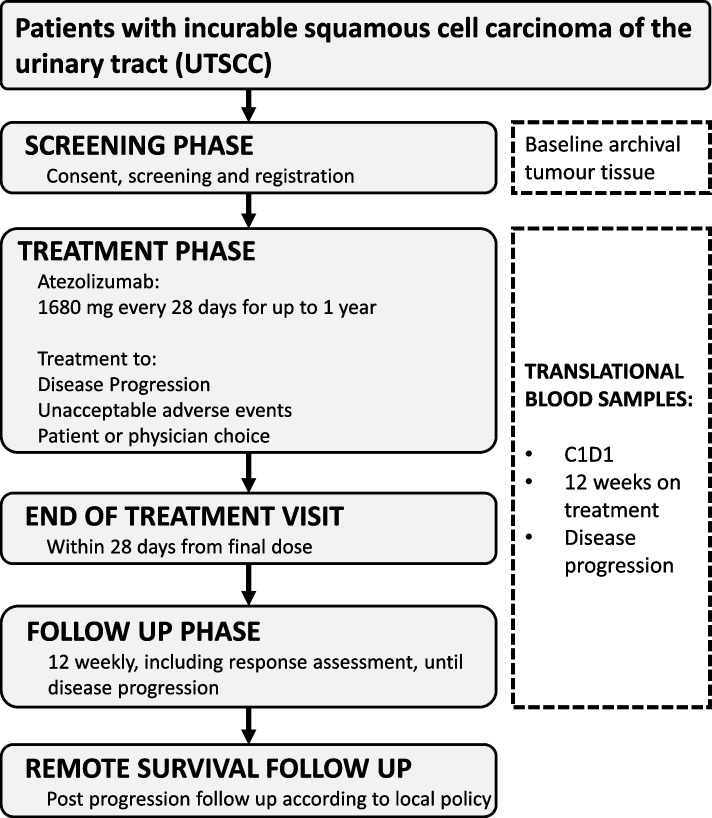
AURORA Trial schema

The AURORA trial is a single arm, open-label, multicentre, UK phase II trial of atezolizumab immunotherapy, in immunotherapy naive patients with histologically confirmed UTSCC and without any TCC component. Recruitment is intended to occur over approximately 2 years across approximately 10–15 UK hospitals, with a recruitment target of 33 patients (up to a maximum of 36 patients). Following a Screening Phase of up to 28 days, eligible patients will be registered and will commence atezolizumab immunotherapy, every 28 days, within a Treatment Phase of up to 1 year. On treatment discontinuation, patients will be reviewed at an End of Treatment Visit, and then 12 weekly (timed such as to continue with the 12 weekly schedule of CT scans from the Treatment Phase) until disease progression. Following disease progression, patients will revert to routine local follow up processes. Consent will be obtained for long term collection of overall survival status. The AURORA Trial Schema (Fig. 1) details the study design.

#### Treatment

Treatment will consist of atezolizumab, by IV infusion, at a fixed dose of 1680 mg, every 28 days (day 1 of each cycle, ± 3 days), for up to one year. Each participant will receive up to 13 doses in total. If treatment is precluded due to treatment related adverse events, then doses are omitted rather than delayed. Patients will discontinue treatment following disease progression, withdrawal, death or development of significant treatment limiting toxicity. Such patients will continue to be followed up for adverse events, progression and survival in line with the follow up schedule unless they withdraw consent. The Schedule of Events (Table 1) details the trial treatment schedule.

**Table 1:** Schedule of observations and procedures

Visit	Screening Phase	Treatment Phase^a^			End of Treatment Visit	Follow Up Phase	Remote Survival Follow Up^b^
		**Cycle 1** **Day 1**	**Cycle 2** **Day 1**	**Cycle 3 to 13** **Day 1**	**28 days from final dose**	**12 weekly**^**c**^	**As per local policy**
**Week**	**Day -28 to 1**	**1**	**5**	**9 + **			
**Window**			** ± 3 days**	** ± 3 days**	** ± 1 week**	** ± 1 week**	
Informed consent^d^	X						
Inclusion/exclusion criteria	X						
Medical history	X						
Targeted physical exam	X	X	X	X	X	X	
ECOG performance status	X	X	X	X	X	X	
CT chest/abdomen/pelvis^e^	X			X		X	
Biochemistry^f^	X		X	X	X		
Haematology^g^	X		X	X	X		
Archival tumour material	X						
Translational blood samples^h^		X		X^h^	X^h^	X^h^	
Concomitant medications	X	X	X	X	X	X	
Adverse event assessment		X	X	X	X	X	
QOL questionnaire^i^	X			X^i^		X^i^	
Atezolizumab		X	X	X			
Pregnancy Testing (WOCBP)	X^j^	X	X	X			
Patient survival status							X

^a^28-day treatment cycle (± 3 days from last dose)

^b^ Follow up as per local policy

^c^ 12 weekly, until disease progression. Assessment points during the Follow Up Phase should be timed such that they continue seamlessly with the 12-weekly schedule of CT scans during the Treatment Phase

^d^ Consent may be taken up to 2 months prior to Cycle 1 Day 1

^e^ To include cross-sectional imaging of chest, abdomen and pelvis by CT scan during the screening period including response assessment by RECIST, and then every 12 weeks (± 1 week) from enrolment during the Treatment Phase and Follow Up phase until disease progression. (MRI is acceptable if local practice would substitute this, or in cases of renal impairment)

^f^ Serum biochemistry including renal, liver (including ALT, AST, ALP and bilirubin), bone (including serum albumin and calcium) and thyroid profiles, and random cortisol and glucose levels

^g^ Including Hb, WCC, neutrophil count, platelet count and differential

^h^ Translational blood sample for C1D1, C4D1 (12 weeks on treatment) and at disease progression only. Samples may be collected up to 1 week prior to IMP treatment

^i^ Quality of life questionnaire (EORTC QLQ-C30) to be completed during screening and every 12 weeks to disease progression

^j^ Pregnancy test at Screening must be serum test. All pregnancy testing following this can be urine testing

Prohibited concomitant medications include immunotherapy other than atezolizumab, systemic cancer therapy not specified in the protocol, investigational agents not specified in the protocol and radiation therapy (need for palliative radiation therapy should be discussed with the Sponsor but would be likely to require discontinuation of atezolizumab on the grounds of disease progression). Corticosteroids for purposes other than to modulate symptoms from an adverse event related to suspected immunological aetiology, or for cancer complications (e.g., for spinal cord compression), should be discussed with the sponsor. Need for corticosteroidsfor cancer complications would be likely to require discontinuation of atezolizumab on the grounds of disease progression. Use of inhaled corticosteroids, physiologic replacement doses of corticosteroids (i.e., for adrenal insufficiency), and mineralocorticoids (e.g., fludrocortisone) is allowed.

For suspected immune-related adverse reactions, thorough evaluation to confirm aetiology or exclude other causes will be performed. Based on the severity of the adverse reaction, atezolizumab will be withheld and corticosteroids administered. Immune-related adverse reactions that cannot be controlled with systemic corticosteroid use will be reviewed for consideration of administration of other systemic immunosuppressants. Toxicities associated, or possibly associated, with atezolizumab treatment will be managed according to standard procedures for administration of this agent at the treating site. Guidance on dose modification in relation to specific treatment related toxicities is also provided within the Investigators Brochure. Atezolizumab will be permanently discontinued for any Grade 3 immune-related adverse reaction that recurs and for any Grade 4 immune-related adverse reactions, except for endocrinopathies that are controlled with replacement hormones. There will be no dose reductions for atezolizumab. Where required, excessive treatment related toxicity will be managed by periods of dose omission rather than dose delay. As such, there will be no extension to the planned total duration of treatment of 1 year (up to 13 doses at 28-day intervals). If corticosteroids are initiated for treatment of toxicity, it is recommended that they are tapered over ≥ 1 month to the equivalent of ≤ 10 mg/day oral prednisolone before atezolizumab is resumed. Atezolizumab may be omitted for ≤ 12 weeks to allow for improvement in toxicity and for patients to taper off corticosteroids prior to resuming treatment. If atezolizumab is withheld for > 12 weeks after event onset, the patient will be permanently discontinued from atezolizumab irrespective of toxicity severity.

#### Setting

AURORA will be run in 10–15 secondary care hospitals in the UK with the aim of recruiting 33 patients.

#### Sample size and recruitment

This study uses a modified Simon’s 2-Stage optimal design with best Overall Response Rate (ORR; % with confirmed partial or complete response by RECIST v1.1) at a minimum of 12 weeks from commencing treatment as the primary endpoint. We propose that an ORR of < 15% would not warrant further investigation, and an ORR ≥ 35% would warrant further investigation in a randomised trial. With a one-sided Type I error rate of 0.1, 90% power, p1 = 15% and p2 = 35%, up to 19 patients will be recruited into Stage 1. If 4 or more patients out of the 19 in Stage 1 (with a minimum of 12 weeks from commencing treatment) have an objective response (confirmed partial response (PR) or complete response (CR) by RECIST v1.1) then the trial will continue with ongoing recruitment to Stage 2 recruiting a maximum of 14 further patients or 33 patients overall. There is no pause in recruitment whilst the Stage 1 analysis is undertaken. Otherwise, accrual will close. If 8 or more patients overall have a best objective response by RECIST (confirmed PR or CR) then this intervention will be considered to have clinical activity that warrants further investigation. To account for drop out, prior to commencing treatment, up to an additional 3 patients may be recruited, making a possible final sample size of 36 patients.

The trial will aim to open sites with a good geographical spread across the UK and encourage cross-referrals between hospitals to minimise the challenges of recruiting in rare disease groups.

### Ethical and regulatory aspects

The study received ethical approval from Chelsea Research Ethics Committee on 25-May-2022 (ref: 22/LO/0272) and has Health Research Authority (IRAS 1004493) and UK Medicines and Health Care Product Regulatory Agency (MHRA) approvals. Southampton Clinical Trials Unit (SCTU), a Cancer Research UK core funded and UK Clinical Research Collaboration registered Clinical Trials Unit (CTU), is coordinating the trial.

### Study participants

The AURORA trial is currently recruiting patients with histologically confirmed urinary tract squamous cell carcinoma (UTSCC), without any transitional cell carcinoma (TCC) component. Patients will have newly diagnosed or progressive measurable disease (as defined by RECIST v1.1), without prior systemic immunotherapy and patients should not be suitable for curative treatment. Up to one prior line of systemic chemotherapy will be acceptable. The full eligibility criteria are listed in Table 2.

**Table 2:** AURORA trial eligibility criteria

**Inclusion criteria**	
1	Histologically confirmed cancer of the urinary tract with squamous cell carcinoma histology and without any TCC component. Mixed non-TCC histology is allowed if squamous cell carcinoma is the predominant histology
2	Newly diagnosed or progressive measurable disease as defined by RECIST version 1.1. To be considered measurable (and to be designated as a target lesion), a lesion must not have been treated with prior radiotherapy or focal ablation techniques, unless disease progression has been subsequently demonstrated in that lesion by an increase in its diameter of at least 20% in long axis (or short axis for lymph nodes)
3	Suitable, in the judgment of the local investigator, for treatment with atezolizumab, with palliative intent
4	Adequate haematologic and end-organ function within 28 days prior to the first study treatment including: a) Absolute neutrophil count ≥ 1.5 × 10^9^/L b) Platelet count ≥ 100 × 10^9^/L c) Haemoglobin ≥ 90 g/L d) Aspartate transaminase (AST), alanine transaminase (ALT), and alkaline phosphatase ≤ 2.5 times the institutional upper limit of normal (ULN) e) Total bilirubin ≤ 1.5 times ULN (or ≤ 3 ULN in patients with Gilbert’s syndrome) f) Calculated creatinine clearance ≥ 20 mL/min (Cockcroft-Gault formula)
5	Up to one prior line of systemic chemotherapy for UTSCC. Neoadjuvant/adjuvant chemotherapy that was completed (from the date of the last dose) more than 12 months prior to progression of disease, or systemic chemotherapy provided as a radio-sensitising component of chemo-radiotherapy (given at any time), do not count as a prior line of treatment for these purposes. In addition where cisplatin was switched to carboplatin, for example due to toxicity, during the course of treatment this will not count as a separate line of therapy
6	Eastern Cooperative Oncology Group (ECOG) performance status of 0 to 2
7	Life expectancy ≥ 12 weeks
8	Representative formalin-fixed paraffin embedded (FFPE) tumour sample with an associated linked-anonymised pathology report that is available for central use in translational studies
9	Able to comply with all trial procedures and processes
10	Age ≥ 18 at time of signed inform consent form
11	Provision of written informed consent
**Exclusion criteria**	
1	Any component of TCC histology
2	Planned for treatment with curative intent
3	Prior systemic immunotherapy (prior intra-vesical treatments are allowed)
4	Major surgery within 30 days prior to enrolment
5	History of severe allergic, anaphylactic, or other hypersensitivity reactions to chimeric or humanized antibodies or fusion proteins
6	Known hypersensitivity to biopharmaceuticals produced in Chinese hamster ovary cells or any component of the atezolizumab formulation
7	Use of oral or IV steroids for 14 days prior to enrolment. Use of inhaled corticosteroids, physiologic replacement doses of glucocorticoids (i.e., for adrenal insufficiency), and mineralocorticoids (e.g., fludrocortisone) is allowed
8	Administration of a live or attenuated vaccine within 4 weeks prior to enrolment (COVID-19 vaccination is allowed)
9	Treatment with any other investigational agent within 4 weeks prior to enrolment
10	Coronary artery bypass graft, angioplasty, vascular stent, myocardial infarction, unstable arrhythmias, unstable angina or congestive cardiac failure (New York Heart Association ≥ grade 2) within 6 months prior to enrolment
11	Patients with known HIV infection or with active tuberculosis
12	Patients with known active hepatitis B virus (HBV; chronic or acute; defined as having a positive hepatitis B surface antigen [HBsAg] test) or hepatitis C. Patients with past HBV infection or resolved HBV infection (defined as the presence of hepatitis B core antibody and the absence of HBsAg) are eligible. Patients positive for hepatitis C virus (HCV) antibody are eligible only if polymerase chain reaction is negative for HCV RNA
13	Autoimmune disease including myasthenia gravis, myositis, autoimmune hepatitis, systemic lupus erythematosus, rheumatoid arthritis, inflammatory bowel disease, vascular thrombosis associated with antiphospholipid syndrome, Wegener’s granulomatosis, Sjögren’s syndrome, Guillain-Barré syndrome, multiple sclerosis, vasculitis, or glomerulonephritis. Patients with a history of autoimmune-related hypothyroidism on a stable dose of thyroid replacement hormone or with controlled Type I diabetes mellitus on a stable dose of an insulin regimen are eligible for this study
14	History of idiopathic pulmonary fibrosis, organizing pneumonia (e.g., bronchiolitis obliterans), drug-induced pneumonitis, idiopathic pneumonitis, or evidence of active pneumonitis on screening chest CT scan. A history of radiation pneumonitis in the radiation field (fibrosis) is permitted
15	Prior allogeneic stem cell or solid organ transplant
16	Patients who are pregnant or breast feeding
17	Patients of child-bearing potential who are not able to use a highly effective method of contraception
18	A recent or current other cancer. Current non-melanoma skin cancer, cervical carcinoma in situ or localized prostate cancer not requiring current treatment are permissible, as is a history of a separate other malignancy having completed all active treatment ≥ 2 years previously

#### Withdrawal criteria

Participants are free to withdraw consent from the study at any time without providing a reason. Where possible, participants who have withdrawn from trial treatment should remain in follow-up as per the trial schedule. Patients who withdraw from treatment only will continue to have assessments until disease progression. Data and samples collected prior to participant withdrawal will still be used for trial analysis.

### Study procedure

#### Informed consent

Consent to enter the trial will be sought from each participant only after a full explanation has been given, a participant information sheet (PIS) offered and allowed time for consideration. Signed participant consent will be obtained using the trials Informed Consent Form (ICF). Consent will be documented in the patient’s medical records. Only site staff authorised on the Delegation Log may obtain consent. Patients may refuse to participate without giving reasons and this will not prejudice their future treatment. The trial’s PIS and ICF detail the consent provisions for collection and use of participant data and biological specimens in future research.

#### Screening

Following informed consent for the main trial, screening assessments should be completed within 28 days prior to commencing treatment. These include a CT scan being undertaken with RECIST v1.1 assessment (MRI is acceptable if local practice would substitute this, or in cases of renal impairment), physical examination, ECOG performance status, biochemistry (including renal, liver, bone and thyroid profiles, plus random cortisol and glucose levels) and full blood count. Concomitant medication and medical history will be recorded, and a quality of life (QoL) questionnaire will be completed. Archival tumour sample should be provided to the trials tissue bank. In addition, women of childbearing potential (WOCBP) will undertake a pregnancy test.

#### Treatment and follow-up visits

Participants will receive atezolizumab for a study period up to 1 year, consisting of 13 × 28-day cycles. Participants will be treated until disease progression, withdrawal, death or development of significant treatment limiting toxicity. Patients will attend hospital appointments for treatment cycles with assessments similar to those performed during screening, plus reviews of adverse events and treatment adherence. The time schedule of enrolment, interventions, assessments, and visits for participants are fully detailed in the schedule of events (Table 1). Participants will receive CT scans, including response assessment by RECIST v1.1, every 12 weeks. An End of Treatment visit will be completed 28 days after the participants final dose, with assessments similar to those performed during treatment. Participants completing the treatment phase will continue to be followed up every 12 weeks until disease progression or the end of the trial. For patients that experience disease progression (during treatment or follow-up), patients will revert to standard of care, as per local practice. Survival status will be collected for all patients when all patients have been followed up for a minimum of 12 months (or have experienced disease progression if this happens sooner). Serious adverse events (SAEs) and adverse events of special interest (AESI) will be reported in real-time to the SCTU safety desk throughout the study. SAEs are assessed to determine relatedness and expectedness, and will be reported to Roche and the relevant UK regulatory bodies.

#### Translational research

Archival fixed paraffin embedded (FFPE) tumour samples will be collected at baseline on all patients. Availability of an archival tumour sample, with an associated linked-anonymised pathology report, for transfer for use in the AURORA trial will be an eligibility requirement for trial entry. Blood samples will be collected in EDTA vacutainers, Streck tubes and PAXgene tubes at baseline (cycle 1, day 1), at 12 weeks on treatment (cycle 4, day 1) and at the point of disease progression in all patients. All samples (PBMCs, plasma and whole blood) will be registered with the UKCRC Tissue Directory.

#### Contraception

For women of childbearing potential, two highly effective forms of contraception must be used. Male patients with partners of child-bearing potential should agree to take measures not to father children by using one form of highly effective contraception. Male participants must also refrain from donating sperm during this period. Men with pregnant or lactating partners must be advised to use barrier method contraception (for example: condom plus spermicidal gel) to prevent exposure to the foetus or neonate.

### Data collection and management

#### Plans for assessment and collection of outcomes

Hospital research staff will enter participant data into the study electronic case report forms (eCRFs) via a remote data collection tool (Medidata Rave). Only trained personnel with specific roles in the study will be granted access to the eCRFs. SCTU trial staff will regularly check the data for missing or anomalous values. Data queries will either be automatically generated within the eCRF, or manually raised with site by the SCTU team. Site staff will respond to explain or resolve the discrepancies.

The PIS and ICF will outline the participant data to be collected and how it will be managed or might be shared, including handling of all Patient Identifiable Data (PID) and sensitive PID adhering to relevant data protection law.

#### Data management

Participant data will be entered remotely at site and retained in accordance with the current Data Protection Regulations. The Principal Investigator (PI) at each site is responsible for ensuring the accuracy, completeness, and timeliness of the data entered. Only the investigator and personnel authorised by them should enter or change data in the eCRFs.

The participant data is pseudo anonymised by assigning each participant a participant identifier code which is used to identify the participant during the trial. The site retains a participant identification code list, which is only available to site staff.

Data queries will either be automatically generated within the eCRF, or manually raised by the SCTU, if required. All alterations made to the eCRF will be visible via an audit trail.

At the end of the trial, after all queries have been resolved and the database frozen, the PI will confirm the data integrity by electronically signing all the eCRFs. The eCRFs will be archived according to SCTU policy and a PDF copy including all clinical and Meta data returned to the Investigator for each participant.

### Oversight and monitoring

The AURORA Trial Management Group (TMG) is responsible for overseeing the progress of the trial, including both the clinical and practical aspects. The Chair of the TMG is the Chief Investigator of the trial. The TMG includes representatives with expertise in oncology; as well as being supported by two Patient and Public Involvement contributors and CTU staff involved in the day-to-day running of the trial. An independent Trial Steering Committee (TSC) has also been established, and an independent Data Monitoring and Ethics Committee (DMEC) comprising two clinicians and a statistician experienced in this research area (but not otherwise involved in this trial) has been set up. The aim of the independent DMEC is to safeguard the interests of trial participants, monitor the main outcome measures including safety and efficacy, and monitor the overall conduct of the trial. Charters for these groups are available via AURORA@soton.ac.uk.

The trial is also subject to inspection and audit by University Hospital Southampton NHS Foundation Trust (as Sponsor), SCTU (as the Sponsor’s delegate) and other regulatory bodies to ensure adherence to the principles of GCP, Research Governance Framework for Health and Social Care, applicable contracts/agreements and national regulations.

For site monitoring, the PI will allow the SCTU direct access to relevant source documentation for verification of data entered onto the eCRF (taking into account data protection regulations). Monitoring procedures are fully described in a trial monitoring plan. Participants’ medical records and other relevant data may also be reviewed during audit of the trial. Details will remain confidential and participants’ names will not be recorded outside the trial site without informed consent.

### Statistical analysis

A detailed statistical analysis plan will be developed prior to database lock, and all data and appropriate documentation will be stored for a minimum of 25 years after the completion of the trial.

The primary analysis of best ORR will be based on the principal stratum strategy where patients who do not receive at least one dose of study treatment will be excluded from the analysis and replaced. This population will be known as the evaluable population. The best ORR will be calculated as the percentage of patients who achieve either PR or CR according to RECIST v1.1. Patients who do not have a 12-week on-treatment response assessment due to drop out will be considered as treatment failure. Therefore, we’ll have a composite endpoint of best overall response rate or failure due to drop out. A 95% confidence interval for the proportion will be calculated. A secondary analysis will be performed according to the intention to treat (ITT) principle, with treatment-related intercurrent events (non-initiation, missed doses, early withdrawal) handled using the treatment strategy policy.

For the secondary endpoints, the time-to-event endpoints of PFS and OS will be analysed and presented using Kaplan–Meier curves for the ITT population will be displayed. The 95% confidence intervals for median survival will be computed using the method of Brookmeyer and Crowley. Response data (according to RECIST v1.1) will be presented using waterfall, spider and swimmers plots with all other analyses being descriptive (i.e., medians and IQRs, means and 95% CI and proportions with 95% CI). All adverse events (AEs) and serious adverse events (SAEs) by relatedness will be listed and summarised by system/organ class, grade (CTCAE v5.0) and term (MedDRA) on the safety population (those receiving at least one dose of study drug). We will assess treatment compliance by summarising the percentage of received dose relative to intended dose.

All analyses will be carried out using STATA 16 or higher and/or SAS 9.4 or higher.

### Interim analysis

The DMEC will review the emergent response data from the trial as recruitment progresses, which will be used to make a recommendation on whether to transition from Stage 1 to Stage 2. The Stage 1 interim analysis will occur once 19 patients are recruited and each have reached at least the first 12-week on-treatment response assessment (CT scan) point, or have unequivocal disease progression at an earlier point. Patients withdrawing earlier than the planned week-12 response assessment will be assessed as non-responders for the interim analysis. To allow for late responses occurring beyond 12 weeks, the DMEC will have the option to defer a decision on proceeding from Stage 1 to Stage 2 until all Stage 1 patients have completed 12 months of follow up if this would potentially alter this decision.

The trial will continue recruitment during the Stage 1 interim analysis period to optimise the recruitment rate to the trial. The justification for this is that atezolizumab has a well-established safety and toxicity profile and this is a disease setting with no established alternative treatment options. Patients will be informed of this approach as part of the informed consent process. The DMEC and Trial Management Group will have the option to integrate a recruitment pause between Stage 1 and Stage 2 if they deem this appropriate based on emergent results.

### Adverse event reporting and risks 

Data on AEs will be collected at treatment and follow-up visits from cycle 1 day 1 until 30 days post treatment. SAEs will be reported from cycle 1 day 1 until 90 days post treatment. AEs and SAEs considered by the investigator to be related to trial procedures will also be collected between consent and cycle 1 day 1. The SCTU has a UK regulatory compliant real-time SAE reporting process to identify serious AEs and suspected unexpected SAEs that could suspend or stop the trial if warranted.

Participants will have CT scans at regular intervals throughout the trial to monitor the extent of the disease following RECIST v1.1. The frequency of these scans generally match standard of care in these patients, but patients will receive one additional scan when taking part in the trial. Patients will be made fully aware of this and the risks of these scans prior to giving consent to their participation in the trial.

Whilst patients eligible for participation in AURORA may have already been treated with an approved systemic chemotherapy for UTSCC, there is no current data to support use of immunotherapy for UTSCC. Therefore, patients are not excluded from any standard treatment by participating in the trial. Participants in AURORA will be treated with atezolizumab, which has demonstrated efficacy in patients with advance TCC histology urinary tract cancer [10–12]. Patients treated with atezolizumab may experience toxicity due to these treatments. However, toxicity and patient safety will be closely monitored. The non-clinical safety profile and emerging clinical profile, from studies using these drugs, have not identified risks that would preclude investigation in this setting. Finally, although there can be no certainty of clinical benefit to patients, it is hoped that atezolizumab will improve outcomes for this group of patients. Trial safety data will be closely reviewed by the DMEC. Considering all these factors, the risk–benefit assessment for patient participation in the AURORA trial is assessed to be favourable as the treatment has the potential to offer a new and safe treatment strategy for patients with UTSCC.

### End of the trial

All patients will be followed up for a minimum of 12 months after the last patient is recruited (or all patients have experienced disease progression if this happens sooner). End of Trial is defined as when the last patient has had their last trial visit and all data to answer the research objectives have been collected.

## Discussion

AURORA aims to determine the clinical activity of atezolizumab in patients with incurable UTSCC. If AURORA can demonstrate that atezolizumab is both effective and safe, it may lead to a future randomised trial run across the International Rare Cancer Initiative (IRCI) partnership with the aim to create evidence for a systemic treatment option for a patient population that currently lack options and are often excluded from trial participation. Results of AURORA will be disseminated to patients and clinical teams through peer-reviewed journal publications and by engaging with relevant patient organisations.

## Trial status

The trial opened to recruitment on 30-May-2022. 33 patients will be recruited over a 24 month recruitment period at approximately 10–15 sites in the UK.

This clinical trial was entered into EudraCT on 8^th^ September 2021 (2021-001995-32) and registered on ClinicalTrials.gov on 9^th^ September (NCT05038657) and ISRCTN on 3^rd^ November 2022 (ISRCTN83474167). The current protocol is version 5 dated 15-JUN-2023. REC/MHRA approved protocol amendments are communicated to sites via email and updated trial documentation provided centrally via the trial website. Trial registries will be amended where relevant with explanations for these changes.

## Data Availability

Pseudonymised individual participant data within the clinical trial dataset will be available for sharing via controlled access by authorised SCTU staff (as delegated to SCTU by the trial sponsor). Data access can be requested via a SCTU Data Release application form; detailing the specific requirements and the proposed research, statistical analysis, publication plan and evidence of research group qualifications. Please email the completed form to the SCTU Data Release Committee Coordinator at ctu@soton.ac.uk. Data access requests are reviewed against specific eligibility criteria by the SCTU data sharing committee including PPI representation and key members of the trial team. Decisions about requests are made promptly and usually no more than three months after receipt of request. Responses to all data requests, with a clear rationale for any refusals, will be sent promptly to the data requester.

## Abbreviations

AE: Adverse Event
AESI: Adverse Event of Special Interest
CRUK: Cancer Research UK
CTCAE: Common Terminology Criteria for Adverse Events
CTU: Clinical Trials Unit
GCP: Good Clinical Practice
IB: Investigator Brochure
DMEC: Data Monitoring and Ethics Committee
eCRF: Electronic Case Report Form
ICF: Informed Consent Form
IMP: Investigational Medicinal Product
ITT: Intention To Treat
MedDRA: Medical Dictionary for Regulatory Activities
MHRA: Medicines and Healthcare products Regulatory Agency
OS: Overall Survival
ORR: Overall Response Rate
PR: Partial Response
PFS: Progression Free Survival
PIS: Participant Information Sheet
REC: Research Ethics Committee
SAE: Serious Adverse Event
SCTU: Southampton Clinical Trials Unit
TMG: Trial Management Group
TCC: Transitional Cell Carcinoma
TSC: Trial Steering Committee
UTSCC: Urinary Tract Squamous Cell Carcinoma
WOCBP: Woman Of Child Bearing Potential
